# Comparative studies on phenolic profiles, antioxidant capacities and carotenoid contents of red goji berry (*Lycium barbarum*) and black goji berry (*Lycium ruthenicum*)

**DOI:** 10.1186/s13065-017-0287-z

**Published:** 2017-06-24

**Authors:** Tahidul Islam, Xiaoming Yu, Tanvir Singh Badwal, Baojun Xu

**Affiliations:** 1 Food Science and Technology Program, Beijing Normal University-Hong Kong Baptist University United International College, 28, Jinfeng Road, Tangjiawan, Zhuhai, 519085 Guangdong China; 2 Agricultural and Food Engineering Department, Indian Institute of Technology, Kharagpur, West Bengal 721302 India

**Keywords:** Goji berry, Antioxidant, Phenolics, Carotenoids, *Lycium ruthenicum*

## Abstract

**Background:**

The study on phytochemical difference between red and black goji berry is limited.

**Methods:**

Antioxidant activities and phenolic profiles in terms of total phenol content, total flavonoid contents, condensed tannin content, monomeric anthocyanin content, and total carotenoid content of red goji berry (*Lycium barbarum*) and black goji berry (*L. ruthenicum*) were compared using colorimetric assays.

**Results:**

All goji berries were rich in phenolics. Black goji berry had the highest phenolic, condensed tannin content and monomeric anthocyanin content. Black goji berry samples possessed higher antioxidant capacities than red goji berry, while the red goji berry had the highest carotenoid content. Goji berries exhibited a positive linear correlation between phenolic compounds and antioxidant capacities. The average value of carotenoid content in red goji berry was 233.04 µg/g.

**Conclusion:**

The phenolics and antioxidant capacities are much higher in black goji berry than red goji berry, while carotenoid content is much higher in red than black.

## Background

Natural products, in the form of pure compounds or extracts with antioxidant activity, may help the endogenous defense system of the body [1]. Antioxidants obtained through diet are taking on major significance as possible protector agents to diminish oxidative damage. As carcinogenic properties have been reported for some synthetic antioxidants, recent research on the potential applications of natural antioxidants from natural food products, for stabilizing foods against oxidation, have received much attention [2]. Antioxidant supplements or antioxidant containing foods may be used to help the human body to reduce oxidative damage or to protect food quality by preventing oxidative deterioration [3]. The antioxidants contained in foods, especially vegetables, are phenolic compounds (phenolic acids and flavonoids), carotenoids, tocopherol and ascorbic acid [3]. These compounds are important protective agents for human health [4]. Goji berry is a typical example that might be used as nutraceuticals or directly eaten in the diet to maintain good health [5].

Chinese traditional medicinal food goji berry is used for its anti-aging properties, tranquilizing and thirst quenching effects, as well as its ability to increase stamina. The benefits include preventing conditions such as diabetes, hyperlipidemia, cancer, hepatitis, immune disorders, thrombosis, and male infertility [6–8]. There are several clinical and experimental reports showing an anti-diabetic effect of *Lycium barbarum* as it is well-known in traditional Chinese herbal medicine for diabetes. *L. barbarum* reduced oxidation in patients with retinopathy [9]. The presence of various functional components like polysaccharides, flavonoids and carotenoids in *L. barbarum* fruits is believed to be responsible for these effects [7, 10, 11]. A group of lipid-soluble compounds is carotenoids with color ranging from yellow to red, have been shown to be present in large quantity in fruits of *L. barbarum* [12]. Several physiological studies have focused on polysaccharides and carotenoids; however, flavonoids have been less investigated, especially for their antioxidant activity [13, 14]. *L. barbarum* fruit and polysaccharide from it possess a range of biological activities, including anti-aging, neuroprotection, increased metabolism, glucose control in diabetics, glaucoma, anti-oxidant properties, immunomodulation, anti-tumor activity and cytoprotection [13, 15, 16]; *Lycium ruthenicum* fruit contains abundant anthocyanins and a highly branched arabinogalactan protein [17, 18]. Goji berries contain carotenoids (beta-carotene, lutein, lycopene, zeaxanthin, zeaxanthin dipalmitate), polysaccharides (comprising 30% of the pulp), vitamins (ascorbic acid glucopyranosyl ascorbic acid, and tocopherol), fatty acids, betaine, and peptidoglycans [19–22].

As compared to the red goji berry, the study on black goji berry (*L. ruthenicum*) is limited. It is necessary to compare the differences between red and black goji berry in terms of phytochemical and antioxidant capacities. The objectives of the present study aim at assessing the phenolic profile, antioxidant properties and carotenoid content of red goji berry (*L. barbarum*) and black goji berry (*L. ruthenicum*), and provide scientific insight into the phenolic and antioxidant functions of both red and black goji berry to consumers and nutraceutical industry.

## Methods

### Goji berry samples

**Fig. 1:**
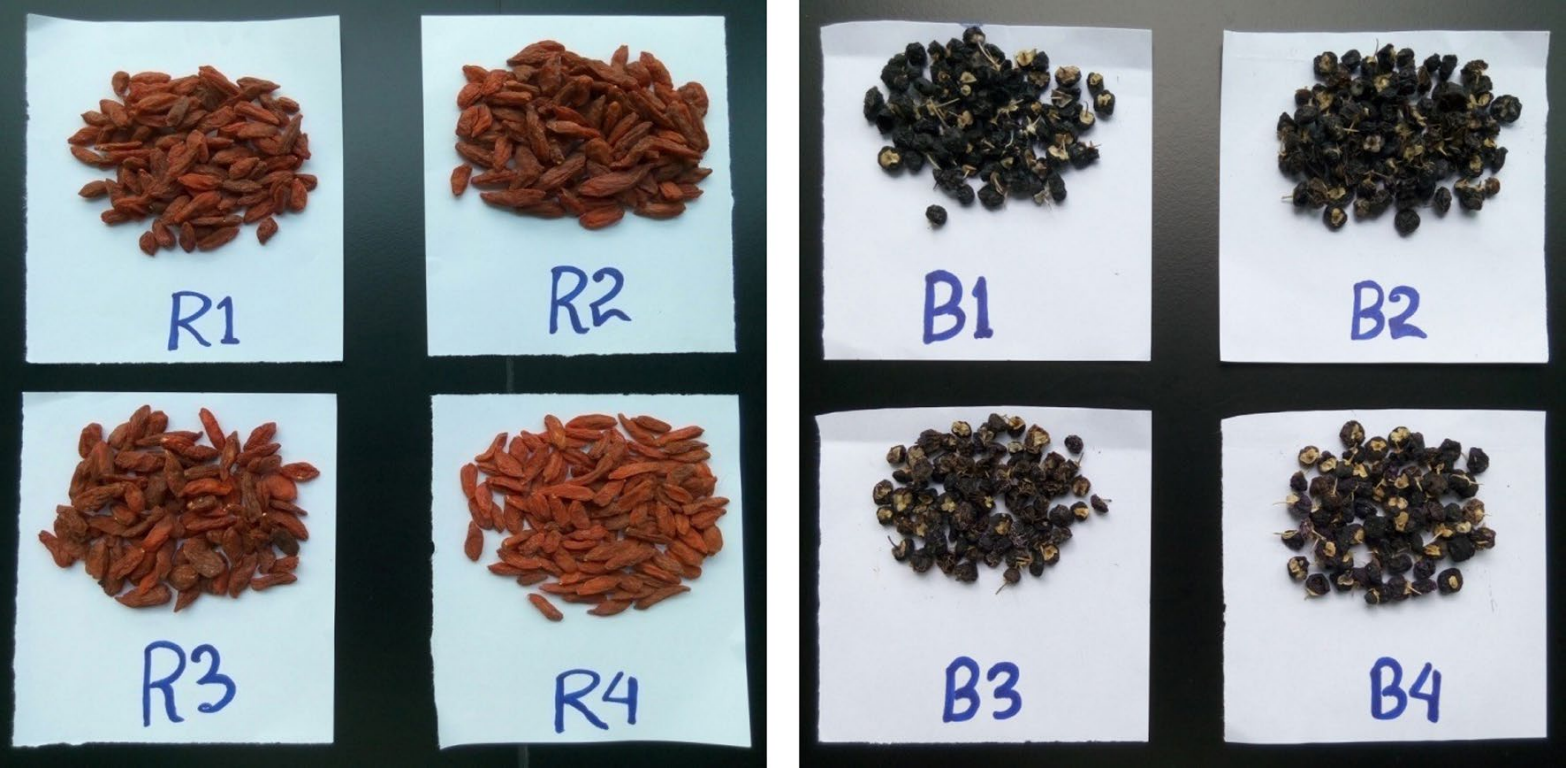
Pictures of red goji berry (*L. barbarum*) and black goji berry (*L. ruthenicum*) fruits

Dried fruits of goji berry (*L. barbarum* and *L. ruthenicum*) belonging to the family of Solanaceae, were produced from Ningxia Autonomous Region and Qinghai Province, China. The sample information is listed in Table 1, and the morphological features based on place of origin of dried goji berry fruits are presented in Fig. 1.

**Table 1:** Sample information of goji berry collected

Sample ID	Common name	Scientific name	Place of origin
R1	Red goji berry	*Lycium barbarum*	LiuYing Village, Xinbao Town, Zhongning County, Zhongwei City, NingXia Hui Autonomous Prefecture
R2	Red goji berry	*Lycium barbarum*	Xinxiaoxian in Xixia District, Yinchuan City, NingXia Hui Autonomous Prefecture
R3	Red goji berry	*Lycium barbarum*	Huangbin Village, Ningan Town, Zhongning County, Zhongwei City, NingXia Hui Autonomous Prefecture
R4	Red goji berry	*Lycium barbarum*	Helan county, Yinchuan City, NingXia Hui Autonomous Prefecture
B1	Black goji berry	*Lycium ruthenicum*	The second battalion of Nuomuhong Farm from Qinghai Province
B2	Black goji berry	*Lycium ruthenicum*	South gate No. 43. Xining City, Qinghai Province
B3	Black goji berry	*Lycium ruthenicum*	Nuomuhong Farm,Qinghai Province
B4	Black goji berry	*Lycium ruthenicum*	The first battalion of Nuomuhong Farm, Qinghai Province

### Chemicals and reagents

2,2′-Azino-bis(3-ethylbenzothiazoline-6-sulfonic acid) (ABTS), Folin–Ciocalteu reagent, 2-diphenyl-1-picryhydrazyl (DPPH), potassium persulphate (K_2_S_2_O_8_), sodium carbonate, gallic acid, sodium hydroxide, sodium nitrite, sodium acetate, acetic acid, hydrogen chloride, 2,4,6-tri(2-pyridyl)-*s*-triazine (TPTZ), ferric chloride, ferrous sulfate, aluminum chloride hexahydrate, (+)-catechin, 6-hydroxy-2,5,7,8-tetramethylchroman-2-carboxylic acid (Trolox), acetone, phosphate buffer saline (PBS), hydrogen chloride (HCl), potassium chloride (KCl), vanillin, methanol, butylated hydroxytoluene (BHT), potassium hydroxide, *n*-hexane was obtained from Sigma-Aldrich Co. (Shanghai, China). Absolute ethanol was from Tianjin Fuyu Fine Chemical Co., Ltd. (Tianjin, China). Other chemical reagents were supplied by Tianjin Damao Chemical Reagent Co., Ltd. (Tianjin, China). All chemicals were of analytical grade unless specially mentioned.

### Extraction of goji berry sample

The goji berry sample extraction procedure was described by Xu and Chang [23]. Briefly, pestle and mortar were used to grind dried goji berry fruits, .5 g of dry ground goji berry samples (in triplicate) were extracted two times with 5 mL extraction solvent of acetone/water/acetic acid (70:29.5:.5) each time. Extracts were shaken for 3 h at 300 rpm using an orbital shaker, then samples extracted were placed in the dark for 12 h. After 12 h the extract samples were centrifuged at 3000 rpm for 10 min. The supernatants were stored at 4 °C in dark for determination of total phenolic content (TPC), total flavonoid content (TFC), total condensed tannin content (CTC), monomeric anthocyanin content (MAC), and antioxidant activities.

### Determination of TPC

Total phenolic content was determined using a colorimetric method as described by Singleton et al. [24, 25]. The absorbance was measured by a UV–visible spectrophotometer (TU-1901) at 765 nm. The TPC was expressed as gallic acid equivalents (mg GAE/g sample) in accordance to standard calibration curve of gallic acid with linear range of 50–1000 µg/mL (R^2^ > .99).

### Determination of TFC

Total flavonoids content was determined using a colorimetric method as described by Heimler et al. [26]. The absorbance was measured by a UV–visible spectrophotometer (TU-1901) at 510 nm. The TFC was expressed as catechin equivalents (mg CAE/g sample) in accordance to standard calibration curve of catechin with linear range from 10 to 1000 µg/mL (R^2^ > .99).

### Determination of CTC

Condensed tannin content was determined using a colorimetric method as described by Broadhurst and Jones [27]. The absorbance was measured by a UV–visible spectrophotometer (TU-1901) at 500 nm. The CTC was expressed as catechin equivalents (mg CAE/g sample) in accordance to standard calibration curve of catechin with linear range of 50–1000 µg/mL (R^2^ > .99).

### Determination of MAC

Monomeric anthocyanin content monomeric anthocyanin content was based on a pH differential method described previously by Lee et al. [28] with no modifications. The MAC was calculated in the form of w/w % of total anthocyanin in the samples using the molecular weight for cyanidin-3-glucoside (449.2 g/mol) and its extinction coefficient (26,900 L cm/mol). MAC was expressed as cyanidin-3-glucoside equivalents because of its historical usage for similar assays and its wide commercial availability [28].

### Determination of DPPH free radical scavenging capacity

DPPH was determined using a colorimetric method as described by Chen and Ho [29]. The absorbance was measured by a UV–visible spectrophotometer (TU-1901) at 517 nm using extraction solvent to replace the sample as blank. The DPPH was expressed as Trolox equivalents (µmol TE/g sample) according to standard calibration curve of Trolox with a linear range from 100 to 750 µM (R^2^ > .99).

### Determination of Ferric reducing antioxidant capacity

Ferric reducing antioxidant capacity (FRAP) was determined using a colorimetric method as described by Benzie and Strain [30]. The absorbance was measured by a UV–visible spectrophotometer (TU-1901) at 593 nm using extraction solvent to replace the sample as blank. The FRAP value was expressed as mmol of Fe^2+^ equivalents per 100 g of sample (mmol Fe^2+^ E/100 g sample) according to standard calibration curve of Fe^2+^ with linear range from 50 to 1000 µM (R^2^ > .99).

### Determination of ABTS radical scavenging assay

ABTS was determined using a colorimetric method as described by Brown and Miller [31], and Re et al. [32]. The absorbance was measured by the UV–visible spectrophotometer (TU-1901) at 734 nm after 6 min reaction in spectrophotometer set at 30 °C, extraction solvent used as blank. The ABTS value was expressed as Trolox equivalents (µmol TE/g sample) in accordance to standard calibration curve of Trolox with linear range from 50 to 1000 µM (R^2^ > .99).

### Determination of total carotenoid content (TCC)

TCC was determined using a colorimetric method as described by Sanusi and Adebiyi [33], with slight modifications. Briefly, a .5 g goji berry sample in triplicates was extracted with 5 mL of ethanolic butylated hydroxyl toluene (ethanol/BHT–100:1, v/w) for isolation and the release of carotenoids. Then, it was mixed completely, and placed in a water bath at 85 °C for 5 min. After that, .5 mL of 80% KOH was added for saponification and properly vortexed before putting it back to 85 °C water bath for 10 min. The mixture was cooled down in an ice-water bath and was added to 3 mL of cold deionized water. Then *n*-hexane (3 mL) was mixed with the mixture before centrifugation at 7500 rpm for 5 min for the separation of two layers. The upper layer with yellow was transferred and collected. This procedure was repeated four times until the upper layers became colorless [34]. Therefore, a total of 12 mL of hexane was put into each centrifuge tube and the final volume of each tube was recorded. The samples were read at the wavelengths of both 450 nm and 503 nm against the hexane as the blank [35]. The concentration of total carotenoid in the extract was calculated by following equation: C_carotene_ = 4.642 × A_450_ − 3.091 × A_503_, where is C concentration of carotenoid expressed in μg/mL, A_450_ = absorbance value at 450 nm, and A_503_ = absorbance value at 503 nm [35]. Finally, the total carotenoid content in dry fruits was expressed in μg/g.

### Statistical analysis

All of the assays were conducted in triplicate extracts and the results were expressed in means ± standard deviations on the basis of dry weight. The significant differences between mean values of samples were determined by analysis of variance (one-way ANOVA) using LSD significant difference test at a significance level of *p* ≤ .05.

## Results

### Total phenolic content of goji berry

The total phenolic contents (expressed in mg GAE/g) of 8 goji berry samples are presented in Table 2. Black goji berry samples B1, B3, B2 and B4 (9.01, 8.95, 8.08 and 7.26 mg GAE/g) had relatively higher total phenolic content, while the red goji berry samples R3, R2, R1, and R4 (2.17, 2.87, 3.12, 4.48 mg GAE/g) had relatively lower phenolic content.

**Table 2:** TPC, TFC, CTC, and MAC of goji berry

Sample no.	TPC (mg GAE/g)	TFC (mg CAE/g)	CTC (mg CAE/g)	MAC (mg/g)
R1	3.12 ± 0.28e	2.67 ± 0.21c	1.24 ± 0.28e	.25 ± 0.98d
R2	2.87 ± 0.28e	2.78 ± 0.21c	1.17 ± 0.28e	.22 ± 0.98d
R3	2.17 ± 1.00f	2.69 ± 0.21c	1.06 ± 0.28e	.21 ± 0.98d
R4	4.48 ± 1.00d	3.16 ± 0.21c	.86 ± 0.28e	.28 ± 0.98d
B1	9.01 ± 0.77a	10.37 ± 0.11b	17.36 ± 1.00d	60.52 ± 1.00c
B2	8.08 ± 1.00b	12.32 ± 0.25a	23.51 ± 1.00a	82.58 ± 0.95a
B3	8.95 ± 0.77a	11.90 ± 0.25a	22.13 ± 1.00b	82.41 ± 0.95a
B4	7.26 ± 1.00c	9.77 ± 0.11b	20.49 ± 1.00c	65.94 ± 1.00b

Data were expressed as mean ± standard deviation (n = 3). The data in the same column marked with different small case letters were significantly (*p* < .05) different

*TPC* total phenolic content, *TFC* total flavonoid content, *CTC* condensed tannin content, *MAC* monomeric anthocyanin content

### Total flavonoid content of goji berry

The total flavonoid contents (expressed in mg CAE/g) of 8 goji berry samples are presented in Table 2. The relatively higher content of flavonoids was recorded in black goji berry samples B2, B3, B1 and B4 (12.32, 11.90, 10.37 and 9.77 mg CAE/g) while the least content of flavonoids was recorded in red goji berry samples R1, R3, R2 and R4 (2.67, 2.69, 2.78 and 3.16 mg CAE/g).

### Total condensed tannin content of goji berry

The total condensed tannin contents (expressed in mg CAE/g) of 8 goji berry samples are presented in Table 2. The relatively higher content of condensed tannin was recorded in black goji berry samples B2, B3, B4 and B1 (23.51, 22.13, 20.49 and 17.36 mg CAE/g) while the least content of condensed tannin was recorded in red goji berry samples R4, R3, R2 and R1 (.86, 1.06, 1.17 and 1.24 mg/g).

### Total monomeric anthocyanin content of goji berry

The total monomeric anthocyanin contents (expressed in anthocyanins mg/g) of 8 goji berry samples are presented in Table 2. Black goji berry samples B2, B3, B4 and B1 (82.58, 82.41, 65.94 and 60.52 mg/g) had relatively higher total phenolic content; while the red goji berry samples R3, R2, R1, and R4 (.21, .22, .25 and .28 mg/g) had relatively lower monomeric anthocyanin content.

### FRAP radical scavenging activity of goji berry

FRAP (expressed in mmol Fe^2+^ E/100 g) of 8 goji berry samples is presented in Table 3. The relatively higher FRAP were recorded in black goji berry samples B3, B2, B1 and B4 (36,346.61, 33,930.79, 28,957.95 and 27,821.53 mmol Fe^2+^ E/100 g), while the least antioxidant capacities were found in red goji berry samples R3, R2, R1 and R4 (2639.03, 3303.13, 3473.79 and 4651.04 mmol Fe^2+^ E/100 g).

**Table 3:** Antioxidant capacities (DPPH, FRAP, ABTS) of goji berry

Sample no.	FRAP (mmol of Fe^2+^ E/100 g)	DPPH (µmol TE/g)	ABTS (µmol TE/g)
R1	3473.79 ± 0.09de	16.07 ± 0.35e	64.38 ± 0.58d
R2	3303.13 ± 0.09de	16.61 ± 0.09de	53.92 ± 0.58f
R3	2639.03 ± 0.28e	16.46 ± 0.09de	55.87 ± 0.08ef
R4	4651.04 ± 0.13d	17.47 ± 0.09c	62.40 ± 0.58de
B1	28957.95 ± 0.13c	35.86 ± 0.74a	150.51 ± 0.33c
B2	33930.79 ± 1.00b	35.68 ± 0.74a	180.03 ± 1.00a
B3	36346.61 ± 1.00a	33.30 ± 0.08b	167.59 ± 1.00b
B4	27821.53 ± 0.13c	32.29 ± 0.08b	147.00 ± 0.33c

Data were expressed as mean ± standard deviation (n = 3). The data in the same column marked with different small case letters were significantly (*p* < .05) different

*FRAP* ferric reducing anti-oxidant capacity, *DPPH* free radical scavenging capacity, *ABTS* radical scavenging assay

### DPPH free radical scavenging activity of goji berry

The DPPH free radical scavenging activity (expressed in µmol TE/g) of 8 goji berry samples is presented in Table 3. The relatively higher DPPH scavenging abilities recorded in black goji berry samples B1, B2 B3 and B4 (35.86, 35.68, 33.30 and 32.90 µmol TE/g) while the least DPPH scavenging abilities were found in red goji berry samples R1, R3, R2 and R4 (16.07, 16.46, 16.61 and 17.47 µmol TE/g).

### ABTS radical scavenging activity of goji berry

The results of ABTS radical scavenging activity of 8 goji berry samples are presented in Table 3. Black goji berry samples B2, B3, B1 and B4 (180.03, 167.59, 150.51 and 147.00 µmol TE/g) exhibited the relatively higher ABTS radical scavenging, while the lowest were found in red goji berry samples R2, R3, R4 and R1 (53.92, 55.87, 62.40 and 64.38 µmol TE/g).

### Total carotenoid content of goji berry

The total carotenoid contents of 8 goji berry samples are presented in Table 4. R1, R3, R4 and R2 (233.08, 224.21, 222.63 and 212.24 µg/g) had the highest carotenoids while the lowest were found in B4, B1, B2, and B3 (1.51, 1.96, 2.77, and 3.19 µg/g).

**Table 4:** Carotenoids (TCC) of goji berry

Sample no.	TCC (µg/g)
R1	233.08 ± 1.00a
R2	212.24 ± 1.00c
R3	224.21 ± 0.61b
R4	222.63 ± 0.61b
B1	1.96 ± 0.62d
B2	2.77 ± 0.62d
B3	3.19 ± 0.62d
B4	1.51 ± 0.62d

Data were expressed as mean ± standard deviation (n = 3). The data in the same column marked with different small case letters were significantly (*p* < .05) different

*TCC* total carotenoids content

## Discussion

### Phenolic compounds in goji berry

The highest TPC value was recorded as 9.01 mg GAE/g while the lowest TPC value was recorded as 2.17 mg GAE/g. The average value of 4 black goji berry samples rich in TPC was recorded as 8.33 mg GAE/g which was 2.6 times higher than the rest 4 red goji berry samples. Average TPC in these 4 red goji berries was recorded as 3.16 mg GAE/g, which differed significantly (*p* < *.05*) from black goji berry. This finding indicates that the goji berry species are a significant source of phenolics.

The highest content of flavonoids was recorded as 12.32 mg CAE/g, while the least flavonoids were recorded as 2.67 mg CAE/g. The average TFC value was recorded as 11.09 mg CAE/g from 4 black goji berry samples, which was 3.9 times higher than the 4 red goji berry samples, the average TFC value of 4 red goji berry samples was 2.83 mg CAE/g, which differed significantly (*p* < .05) from the 4 black goji berry samples.

The highest condensed tannin content was recorded as 23.51 mg CAE/g in black goji berry, while the least condensed tannin content was recorded as .86 mg CAE/g in red goji berry. The tannin content of black goji berry samples 20.87 mg CAE/g, was 19.3 times higher than the 4 red goji berry samples, the average condensed tannin content of 4 red goji berry sample was 1.08 mg CAE/g, which differed significantly (*p* < .05) from the 4 black goji berry samples.

The highest monomeric anthocyanin content was recorded as 82.58 mg MAC/g from black goji berry, while the least condensed tannin content was recorded as .21 mg MAC/g from red goji berry. The average monomeric anthocyanin content was recorded 72.86 mg MAC/g from 4 black goji berry samples, which was 30.4 times higher than the 4 red goji berry samples, the average monomeric anthocyanin content of 4 red goji berry samples was .24 mg MAC/g, which differed significantly (*p* < .05) from the 4 black goji berry samples.

### Antioxidant capacities of goji berry

The highest scavenging activity of goji berry extract was recorded as 35.86 µmol TE/g, while the least DPPH scavenging activity was recorded as 16.07 µmol TE/g. The average value of 4 black goji berry samples was 34.28 µmol TE/g, which was 2 times higher than 4 red goji berries. The average value of 4 red goji berries was 16.65 µmol TE/g.

Table 3 presents the reducing capability of 8 goji berry samples, the highest FRAP value was recorded as 36,346.61 mmol Fe^2+^ E/100 g, and the lowest FRAP value was 2639.03 Fe^2+^ E/100 g. The principle of FRAP assay states that, with reductant (antioxidants) at low pH, ferric tripyridyltriazine (Fe(III)-TPTZ) is reduced to ferrous tripyridyltriazine (Fe(II)-TPTZ) that has an intensive blue color and can be detected at a wavelength of 593 nm [23].

The highest ABTS radical scavenging activity was recorded as 180.03 µmol TE/g from black goji berry, while the lowest ABTS radical scavenging activity was recorded as 53.92 µmol TE/g. The average of 4 black goji berries was 161.28 µmol TE/g, while the lowest value was 59.14 µmol TE/g from 4 red goji berry samples. The ABTS radical scavenging activity is a more sensitive radical that is used for the estimation of antioxidant activity. The reduced ABTS radical is colorless in a color-quenching reaction [36].

### Carotenoid content in goji berry

The total carotenoid contents (TCC) of goji berries are shown in Table 4. The highest carotenoid was 233.08 µg/g from red goji berry, while the lowest value was recorded as 1.51 µg/g from black goji berry. The average value of 4 red goji berries was 223.04 µg/g, while the average value of 4 black goji berries was 2.36 µg/g. The current results are similar as a previous study by Liu et al. [37], in which red goji berry was found to accumulate high levels (a maximum of 508.9 µg/g on fresh weight basis) of carotenoids, while the carotenoids were from 34.46 µg/g to undetectable in the black goji berry.

### Correlation between antioxidant capacities and phenolic compounds

The correlation between antioxidant capacities and phenolics is shown in Table 5. The results of TPC, TFC, CTA, and MAC exhibited positive linear correlation at the level of .01 (r = .5). The results of FRAP, DPPH, and ABTS exhibited a positive linear correlation at the level of .01, where r = .643 for FRAP and DPPH, r = .571 for DPPH and ABTS, and r = .786 for FRAP and ABTS. The correlation between phenolics and antioxidant capacities of 8 goji berry samples exhibited a positive linear correlation at the level of .01, where r = .857 for TPC and FRAP, r = .786 for TPC and DPPH, r = .643 for TPC and ABTS, r = .786 for TFC and FRAP, r = .875 for TFC and DPPH, r = .714 for TFC and ABTS, r = .857 for MAC and FRAP, r = .643 for MAC and DPPH, r = .786 for MAC and ABTS, r = .643 for CTC and FRAP, r = .429 for CTC and DPPH, r = .714 for CTC and ABTS. Between carotenoid (TCC) and phenolics, carotenoid (TCC) and antioxidant capacities of 8 goji berries samples there is a negative correlation. The results dictate that phenolic compounds could be important contributors toward the antioxidant capacities of these goji berries. Phenolic compounds, such as flavonoids, phenolic acids, and condensed tannins, are usually considered to be major contributors to the antioxidant capacities of plants [38].

**Table 5:** Correlation analysis among the antioxidant, phenolics and carotenoids

	TPC	TFC	FRAP	DPPH	ABTS	MAC	CTC	TCC
TPC								
Correlation coefficient	1.000	.643^a^	.857^b^	.786^b^	.643^a^	.714^a^	.500	−.500
Sig. (2-tailed)	–	.026	.003	.006	.026	.013	.083	.083
N	8	8	8	8	8	8	8	8
TFC								
Correlation coefficient	.643^a^	1.000	.786^b^	.857^b^	.714^a^	.786^b^	.571^a^	−.571^a^
Sig. (2-tailed)	.026	–	.006	.003	.013	.006	.048	.048
N	8	8	8	8	8	8	8	8
FRAP								
Correlation coefficient	.857^b^	.786^b^	1.000	.643^a^	.786^b^	.857^b^	.643^a^	−.357
Sig. (2-tailed)	.003	.006	–	.026	.006	.003	.026	.216
N	8	8	8	8	8	8	8	8
DPPH								
Correlation coefficient	.786^b^	.857^b^	.643^a^	1.000	.571^a^	.643^a^	.429	−.714^a^
Sig. (2-tailed)	.006	.003	.026	–	.048	.026	.138	.013
N	8	8	8	8	8	8	8	8
ABTS								
Correlation coefficient	.643^a^	.714^a^	.786^b^	.571^a^	1.000	.786^b^	.714^a^	−.286
Sig. (2-tailed)	.026	.013	.006	.048	–	.006	.013	.322
N	8	8	8	8	8	8	8	8
MAC								
Correlation coefficient	.714^a^	.786^b^	.857^b^	.643^a^	.786^b^	1.000	.786^b^	−.500
Sig. (2-tailed)	.013	.006	.003	.026	.006	–	.006	.083
N	8	8	8	8	8	8	8	8
CTC								
Correlation coefficient	.500	.571^a^	.643^a^	.429	.714^a^	.786^b^	1.000	−.429
Sig. (2-tailed)	.083	.048	.026	.138	.013	.006	–	.138
N	8	8	8	8	8	8	8	8
TCC								
Correlation coefficient	−.500	−.571^a^	−.357	−.714^a^	−.286	−500	−.429	1.000
Sig. (2-tailed)	.083	.048	.216	.013	.322	.083	.138	–
N	8	8	8	8	8	8	8	8

^a^Correlation is significant at the .05 level (2-tailed)

^b^Correlation is significant at the .01 level (2-tailed)

## Conclusions

The 8 black and red goji samples have substantial antioxidant capacity and contain large amount of phenolic compounds. A significant correlation between the DPPH, FRAP and ABTS values suggested that antioxidant assays are reliable. The highly positive correlation between antioxidant capacity, phenolic, flavonoid, condensed tannin and anthocyanin content indicated that phenolic compounds could be the main contributors to the antioxidant activities of these goji berries. The black goji berries have relatively higher antioxidant capacities, total phenolic, flavonoid, condensed tannin and anthocyanin, and it could be an important dietary source of natural antioxidants for the prevention of diseases caused by oxidative stress in human body. This study portrayed an in depth detail on the antioxidant functions of goji berry which is of significant importance to consumers, nutritionists and food researchers.
